# Non-Destructive 3D Elemental Characterization of Multilayer Materials by ANN-Assisted Ion Beam Analysis

**DOI:** 10.3390/ma19132819

**Published:** 2026-07-02

**Authors:** Victoria Corregidor, Nuno P. Barradas, Rui C. da Silva, Teresa Pinheiro, Carlos Algora, Luís C. Alves

**Affiliations:** 1 Departamento de Engenharia e Ciências Nucleares, Instituto Superior Técnico, Universidade de Lisboa, 2695-066 Bobadela, Portugallcalves@ctn.tecnico.ulisboa.pt (L.C.A.); 2 C2TN—Centro de Ciências e Tecnologias Nucleares, Instituto Superior Técnico, Universidade de Lisboa, 2695-066 Bobadela, Portugal; 3 IPFN—Instituto de Plasmas e Fusão Nuclear, Instituto Superior Técnico, Universidade de Lisboa, 1049-001 Lisboa, Portugal; 4 IBB—Instituto de Bioengenharia e Biociências, Instituto Superior Técnico, Universidade de Lisboa, 1049-001 Lisboa, Portugal; 5 Instituto de Energía Solar, Universidad Politécnica de Madrid, Avda. Complutense, 30, 28040 Madrid, Spain

**Keywords:** 3D elemental mapping, layered samples, ion beam analysis, PIXE, EBS, neural networks, non-destructive characterization

## Abstract

**Highlights:**

- ANN-assisted PIXE–EBS enables non-destructive 3D elemental mapping.
- Depth-resolved composition reconstructed from low-statistics spectra.
- Buried elemental distributions revealed beneath thick Au contacts.
- Quantitative 3D mapping achieved with micrometre-scale resolution.
- General framework for multilayer material characterization.

**Abstract:**

Patterned and multilayer materials used in advanced technologies exhibit complex three-dimensional compositional architectures in which buried interfaces and elemental gradients critically influence performance. However, most non-destructive analytical techniques remain largely surface-sensitive, limiting access to subsurface information in opaque systems. In this work, we present a novel framework for non-destructive three-dimensional elemental characterization based on the integration of artificial neural networks with ion beam analysis techniques, namely, Particle-Induced X-ray Emission (PIXE) and Elastic Backscattering Spectrometry (EBS). The proposed approach enables the reconstruction of depth-resolved 3D elemental distributions by combining complementary spectral information with data-driven analysis. The methodology is demonstrated on a GaSb thermophotovoltaic device featuring multilayer metallic contacts, where the elemental distribution beneath thick gold layers is revealed for the first time. The neural network approach overcomes limitations associated with low counting statistics in pixel-resolved spectra, enhancing sensitivity and enabling reliable classification of compositional features. The fusion of PIXE-derived lateral information with EBS-based depth profiling enables full three-dimensional visualization and quantitative and qualitative mapping of elemental distributions. Beyond the specific case study presented, this approach provides a general and scalable strategy for 3D compositional analysis of complex materials, including systems containing both heavy and light elements. The results highlight the potential of combining advanced data-driven methods with ion beam techniques to expand the capabilities of non-destructive characterization, with broad applicability in energy, electronics, and functional materials.

## 1. Introduction

Ion beam analytical (IBA) techniques using MeV protons or alpha particles have many advantages over techniques that use other traditional probes such as electrons or X-rays. Ion beam techniques rely on the nature of ion interactions with matter, such as the low angular spread of the ion beam (the trajectory is a nearly straight line over several microns of material) and the reduced bremsstrahlung background [1], facilitating precise and localized analysis. The simultaneous use of several IBA techniques can achieve accurate depth profiling leading to a complete elemental composition knowledge of the sample, including light elements. The absence of strong multiple scattering and the relatively low damage under controlled fluences make them particularly suitable for non-destructive microanalysis.

In a nuclear microprobe (NM) the ion beam generated by an MV electrostatic accelerator is focused and scanned over the surface of a sample with micrometre lateral resolution in routine operation. Together with the IBA techniques, it allows for immediate 2D elemental distribution maps from regions of interest (ROIs) defined in the IBA spectra and elemental depth profiles; resolution down to tens of nanometres can also be obtained.

Traditionally, individual IBA techniques, such as Particle-Induced X-ray Emission (PIXE) and Elastic Backscattering Spectrometry (EBS), are used for characterization of all types of samples, but what is really powerful and challenging is the combination of different IBA techniques to obtain a univocal composition distribution. This procedure is known as the Total-IBA approach, which has been developed for macro beams and has not been applied for NM 2D data [2,3,4].

NMs also allow, in specific cases, for 3D imaging of metal nanoparticles in light matrix specimens, such as cells, which makes the determination of their location and quantification possible. The pioneering work was published by Chen et al. [5]. They succeeded in obtaining 3D images of gold nanoparticles in whole cells using a 1.6 MeV helium ion beam, combining scanning transmission ion microscopy with forward scattering transmission ion microscopy and EBS. Similarly, Vasco et al. [6] developed a novel software tool, MORIA, to display the distribution of heavy elements (copper oxide nanoparticles) in a light matrix (Saccharomyces cerevisiae cells), leveraging elemental depth distribution from EBS spectra.

Despite these advancements, there are no similar procedures and tools for analysing medium- and high-Z matrices, which are usually found when characterizing some advanced materials, with different applications such as photovoltaics, thermoelectric and other energy-conversion devices. In these materials, compositional inhomogeneities can explain observed experimental variations in energy efficiency, mechanical performance, or thermal conductivity of the final device [7,8,9]. Moreover, the precise quantification of light elements such as C, N and O remains equally challenging, despite their central role in oxides, nitrides, and organic–inorganic hybrid materials [10,11], highlighting the broader need for truly non-destructive 3D compositional mapping techniques.

This challenge is not confined to a single material class: recent studies on quasi-one-dimensional Bi(S,Se)(I,Br) chalcohalides have shown that precise control and mapping of elemental distributions are essential to correlate synthesis parameters with optoelectronic performance [12]. Such results underline the broader need for spatially resolved compositional analysis across functional materials.

Likewise, the energy band gap and lattice parameter of semiconductors, which act as active layers in photovoltaic devices, depend strongly on their composition [13,14,15,16]. V. Corregidor et al. showed that IBA techniques, particularly PIXE and EBS, are suitable to determine the thickness and composition of such active layers in CIGS-based solar cells [17]. In many cases, composition variations can be detected from the classical 2D maps obtained from PIXE spectra, while the in-depth compositional changes are revealed through 2D maps reconstructed from EBS spectra. Thus, the combination of PIXE and EBS can be a real competitive and alternative method to the more commonly used and established techniques such as X-ray fluorescence (XRF) and Energy-Dispersive X-ray Spectroscopy (EDS) for average composition analysis or secondary ion mass spectroscopy (SIMS) for determination of elemental 3D mapping. These IBA techniques have the additional advantage that the experiment is non-destructive and does not significantly alter the composition and structure of the sample.

Here we present, to our knowledge, the first application of ANN-assisted ion beam analysis for non-destructive 3D elemental reconstruction beneath thick metallic contacts in a medium- to high-Z multilayer system. Prior works [18,19,20,21,22,23,24,25,26,27,28] have demonstrated the potential of ANNs for IBA techniques and several recent studies have applied machine learning techniques to ion beam analysis and spectral interpretation. Most studies were on EBS analysis for thin-film systems using neural networks for regression [19,20,21,22,23,24]. Kaspi et al. [25] used a Random Forest algorithm to classify PIXE spectra, without quantitative analysis. Cohen et al. [26,27] used Extreme Gradient Boosting to retrieve elemental amounts from PIXE spectra [27] and to synthesize PIXE spectra from given elemental amounts [26]. All these works operated on spectra from macro-beam measurements, and in no case were 2D or 3D elemental concentrations derived. Tazzioli et al. [28] used an unsupervised learning clustering algorithm for classifying pixels in 2D PIXE/EBS maps with similar characteristics, after which all pixels corresponding to one cluster were added, and the resulting signal was analysed with standard methods. The method did not use ANNs for quantitative analysis and did not provide 3D information. Furthermore, clustering creates an average of regions that share similarities but leads to a loss of detailed 2D information.

The present work differs in three key respects: it operates at the single-pixel level on nuclear microprobe data, where counting statistics per pixel are inherently low; it combines independent PIXE and EBS classifiers in a data-fusion framework to reconstruct full 3D compositional maps; and it targets a system in which conventional 2D IBA mapping cannot access buried layer information. Building on previously developed ANN methodologies for spectral analysis, ANN algorithms are used here as a data-driven tool to extract depth-resolved compositional information from IBA spectra, revealing the 3D distribution of elements buried beneath metallic Au contacts in a GaSb thermophotovoltaic converter [29]—a capability not previously reported for IBA techniques.

3D elemental mapping capabilities are valuable for the development of a wide range of materials, including optical, magnetic and catalytic systems, while also enabling depth-resolved compositional analysis in complex multilayer structures relevant to energy, optoelectronic and functional devices [30,31,32].

## 2. Methods

### 2.1. Thermophotovoltaic Converters

Thermophotovoltaic (TPV) converters were fabricated at Instituto de Energía Solar, Univ. Politécnica de Madrid, Madrid, Spain, on an n-type GaSb substrate using Zn diffusion to form a shallow p–n junction, optimized for infrared radiation absorption. The back contact is formed by evaporation of AuGe/Ni/Au followed by annealing at 250 °C for 1 min.

The front contact is photolithographically defined and consists of 5 nm Cr/25 nm Au/60 nm Ni/1 µm Au, deposited sequentially by thermal evaporation, without any annealing process. While the Cr layer ensures adhesion to the p-GaSb emitter layer and the thin Au/Ni bilayer serves as a diffusion barrier and seed layer, the final 1 µm thick Au cap allows for a suitable wire bonding.

**Figure 1:**
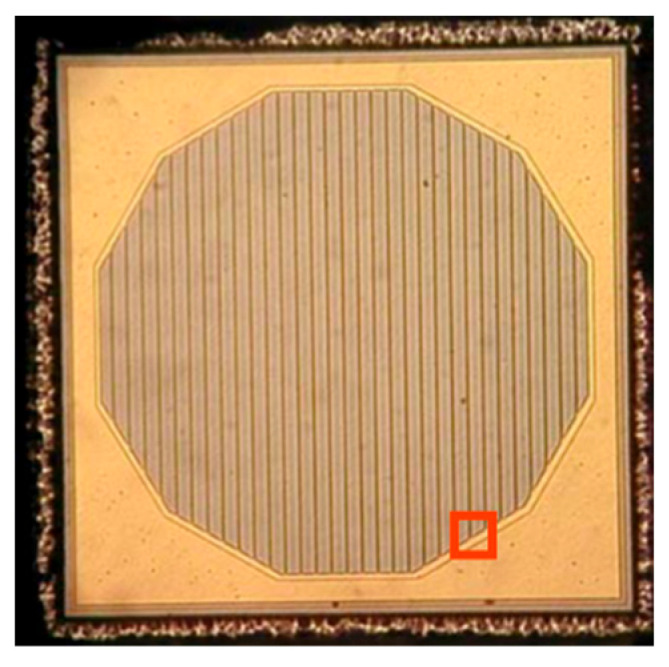
A 2 × 2 mm^2^ GaSb TPV converter. The red square indicates the area that was analysed in detail.

Following contact deposition, individual converters were defined by mesa etching to achieve electrical insulation, resulting in devices with an active area of 2 × 2 mm^2^ (see Figure 1). This structure is designed to operate efficiently under high-temperature thermal emission; fabrication details are reported in [33,34].

### 2.2. IBA Techniques and Nuclear Microprobe

IBA experiments were carried out at CTN/IST (Lisbon, Portugal) using a 2.5 MV Van de Graaff accelerator [35] equipped with an Oxford Microbeams nuclear microprobe (Hampton Poyle, UK) [36,37]. A 2.0 MeV proton beam at normal incidence was used under vacuum conditions, with beam spot dimensions of 3 × 4 µm^2^ and a current of ~100 pA. With these conditions, compositional inhomogeneities can be resolved down to the micrometre scale in depth. The system includes an OM150 magnetic quadrupole triplet (Oxford Microbeams, Hampton Poyle, UK) for beam focusing and an experimental chamber configured for detection of X-rays and backscattered particles, among others, enabling simultaneous PIXE and EBS measurements. X-rays were detected using a 30 mm^2^ SDD detector (Bruker Nano, Berlin, Germany, 145 eV resolution) positioned at 135° relative to the beam axis with a 50 µm Mylar film to stop the backscattered protons. EBS spectra were collected with a 200 mm^2^ PIPS detector (Mirion Technologies, Inc., Atlanta, GA, USA., 30 keV resolution) placed at 140° in Cornell geometry.

Microprobe control and beam scanning were performed using the OMDAQ 3, V. 3.3.4. software. Each scanned area of the sample was acquired as a 256 × 256-pixel matrix map, with each pixel containing the IBA spectra recorded during the experiment. During the measurements, the software allows the user to define regions of interest (ROIs) within the spectra for the generation of detailed maps. For every scanned (x, y) position, the energy signals from the array of detectors surrounding the sample (in this work, the X-ray and the particle detectors) are captured and recorded as a stream of {x, y, E1, …En}-tuples. OMDAQ compiles every recorded data n-tuple into a file known as a listmode file (an event-by-event data collection file), enabling several post-experimental analyses. The listmode file contains information recorded and encoded in binary, and it has an initial block with header information about the operational parameters of the beam and the analogue-to-digital data conversion (ADC). A dedicated code RLModeP [38] was used to read the listmode file and write the spectra from the different detectors, with a user-selected level of pixel compression.

### 2.3. Artificial Neural Network Algorithms

The ANNs were built in Python 3.12 using the TensorFlow library [39], using computational resources from the Cirrus platform of the National Advanced Computing Network in Portugal (RNCA) [40]. Several structures were tested to determine the most suitable for analysing and classifying the IBA spectra. The task was formulated as a classification problem, with the networks trained to assign each spectrum to predefined categories corresponding to elemental composition and gold layer thickness.

A schematic overview of the complete analysis pipeline is provided in the Supplementary Material (Figure S1).

## 3. Data Acquisition and Analysis

**Figure 2:**
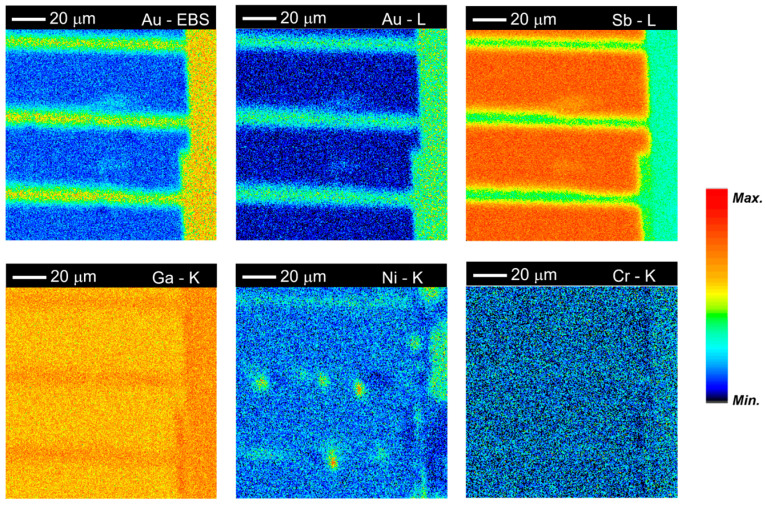
2D elemental maps, 130 × 130 µm^2^ from PIXE spectra recorded during 6 h. For completeness, the gold distribution map obtained from EBS analysis is also shown.

Data from the GaSb-based TPV cell were acquired by scanning the proton beam over 256 × 256 spots corresponding to a 130 × 130 µm^2^ area, generating a listmode file with 65,536 experimental EBS and PIXE spectra. The elemental distribution maps obtained from the PIXE spectra for Au, Ga, Sb, Cr and Ni are shown in Figure 2.

Although the overall EBS and PIXE spectra (obtained by adding all 256 × 256 spectra) may show good counting statistics, the individual spectra from each pixel typically present poor statistics (see, for example, the EBS spectra shown in Figure S2 in the Supplementary Information). As a result, the features and patterns to be identified by ANNs are often difficult to discern. For these reasons, the raw data were compressed by adding each set of 4 × 4 pixels, reducing the number of spectra to 64 × 64 (the 2D elemental maps after this 4 × 4 pixel compression are shown in Figure S3, where it is clear that the resulting lateral resolution is adequate to resolve the features of the region analysed). Furthermore, to increase the statistics, the EBS and PIXE spectra collected with respectively 1024 and 2048 channels, were compressed to 128 and 1024 channels. Each compressed channel had an energy width smaller than a third of the energy resolution FWHM, which is sufficient to discriminate the gold signal in the EBS spectra and resolve the individual X-ray lines in the PIXE spectra. The resulting effective pixel size of ~8 µm is consistent with the beam spot dimensions (3 × 4 µm^2^) and does not compromise the lateral resolution achievable under the experimental conditions used.

The neural networks employed in this work were implemented as classifiers, specifically trained to assign spectra to predefined categories. The classification framework was partitioned into two specific components: a neural network trained for PIXE spectra and another optimized for EBS data. This modular approach enabled independent optimization and facilitated subsequent data fusion and comparative analysis.

For PIXE spectra, in the absence of widely established simulation software to generate the training set needed, alternative approaches were employed. Five representative spectra were selected directly from the elemental distribution maps (see Figure S4) corresponding to distinct regions of the sample that cover the different compositional situations visible across the analysed area. The low background and well-defined elemental peaks make them suitable for classification tasks, reducing the effective dimensionality of the classification problem. Statistical variability was expanded through Poisson-noise augmentation [18,19,20,22], and the resulting validation performance supports the robustness of the classification strategy.

In the future, simulated PIXE spectra may also be generated through linear combinations of reference spectra, as suggested in recent work based on gamma spectra generation [41] or by using Gulys, as suggested by Solis-Lerma et al. [42]. These approaches would further expand the training dataset. At this moment, the total number of spectra generated (adding Poisson noise) was 5000 spectra for each class, splitting them into 80 and 20 for training and validation respectively. To reduce the size of the data generated and the calculation time, a region of interest in the PIXE spectra with 800 channels was selected, covering all the relevant X-ray lines.

Considering the previously presented representative points selected for the PIXE analysis, the corresponding EBS spectra were analysed using the NDF code V.9.6a [43] to calculate the gold layer thickness. Additional spectra were generated using NDF to adequately capture spectral variability associated with variations in the gold layer thickness. This resulted in a representation with eight classes; a classification approach was preferred over regression since discrete thickness bins provide robustness against the statistical fluctuations inherent to low-count individual pixel spectra. From these simulated EBS spectra and adding Poisson noise distribution, thousands of additional training spectra were generated for data augmentation and improved network generalization (10,000 for each class).

As a result, as output data, three variables were defined: the presence of Ni and Cr signals and the thickness of the gold layer on the GaSb substrate. This framework enables the reconstruction of quantitative (Au thickness) and qualitative (Ni and Cr) 3D maps.

The neural network used for PIXE spectra classification was implemented according to the architecture detailed in Supplementary Table S1. It comprises five layers, with an input layer of 800 nodes and an output layer of five nodes. *ReLU* activation functions [44] were applied to the hidden layers, and a *softmax* activation was used in the output layer. Optimization was performed using the *Adam* algorithm [45], and the categorical cross-entropy loss function was employed [46]. The combination of the output activation function with the loss function is appropriate for single-label classification problems, ensuring that each spectrum is assigned to one class only and providing stable convergence during training.

The confusion matrix for the validation set (Figure S5) exhibits a strong diagonal pattern, indicating high classification accuracy and minimal overlap between spectral classes. In this figure, the accuracy as a function of the epoch is also shown. The trained network was subsequently applied to the full set of experimental spectra, none of which were used during training, constituting an independent test of the classification strategy.

Regarding the neural network for the EBS spectra classification, the architecture is in Supplementary Table S2. A fully connected feed-forward neural network was implemented with an input layer of 128 nodes, followed by two hidden layers comprising 96 and 64 neurons with *ReLU* activation functions and an output layer with 8 nodes (corresponding to the number of spectral classes) using a *softmax* activation. The model was compiled with the *Adam* optimizer and categorical cross-entropy loss and trained for 10 epochs with a batch size of 32 using an 80/20 validation split.

For this ANN, the training and validation curves exhibit stable convergence, with both accuracy values exceeding 98% after only a few epochs and minimal divergence between training and validation performance. The accuracy rapidly increases during the first epochs and remains high and consistent thereafter, indicating effective learning and no evident signs of overfitting (Figure S6).

The confusion matrix (Figure S6) for the eight-class EBS spectral classification model shows a strong diagonal dominance indicating high classification accuracy and minimal misclassification between spectral classes, confirming the model’s robust discriminative performance.

## 4. Results

**Figure 3:**
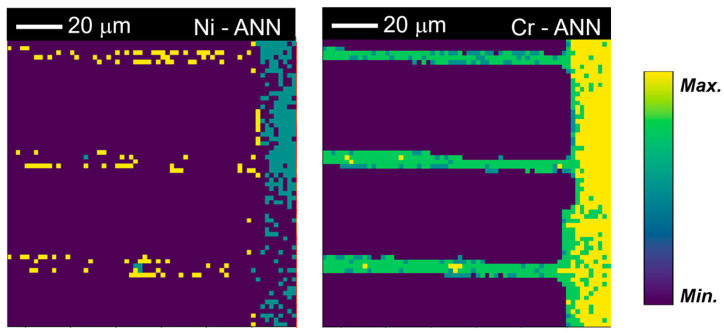
2D maps for Ni and Cr reconstruction from the outputs of the ANN for PIXE spectra.

The reconstructed 2D Ni and Cr elemental distribution maps obtained with the ANN are shown in Figure 3. The ANN-based approach delivered a Cr map with enhanced visual clarity and feature discernibility, superior to that generated with conventional OMDAQ software, as shown in Figure 2, positioning it as a robust alternative to traditional mapping workflows. Continuous Cr coverage is observed, which was not evident from the OMDAQ maps. On the contrary, the Ni map obtained reveals clear structures with discontinuities in the lateral distribution. These findings, which were uniquely obtained with the data processing method now developed, are discussed below.

The results for EBS provided the thickness of the gold contacts. This additional information was fused with the information for Cr and Ni to reconstruct 3D concentration maps, providing access to depth-resolved information not available from 2D mapping alone.

**Figure 4:**
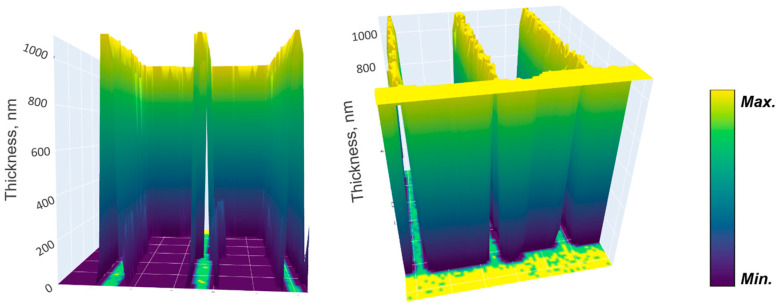
Main 3D view (static rendering) showing the gold layer, and the elemental Ni and Cr distribution (at thickness ~0 nm) corresponding to the Supplementary Video 3D-gold.

Figure 4 combines the information on the spatial distribution of Ni, Cr (shown at the lowest depth, corresponding to thickness ~0 nm), and Au into a single representation, enabling the visualization of their elemental distribution within a three-dimensional environment and providing deeper insight into the sample’s structure beneath the gold contact. Additionally, by performing a three-dimensional analysis of the data (using Python), it is possible to extract the spatially resolved quantitative information corresponding to the concentration and the thickness of the gold layer at each point of the map, enabling spatially continuous thickness mapping across the analysed area (within the assumptions and resolution limits of the IBA techniques). A video illustrating the three-dimensional representation of this information is available in the Supplementary Material.

These three-dimensional maps provide a basis for interpreting the spatial patterns of the constituent elements within the TPV converter, allowing us to relate the visualized distributions to the specific deposition and fabrication characteristics of the front contact described above.

The elements used to form the front contact follow the pattern defined by the photolithographic mask. Some irregularities are found in the gold distribution, which can be related to the inadequate profile of the photoresist during photolithography (i.e., the sidewalls are not sufficiently undercut) or may be due to lift-off conditions, for example. Another feature that can be observed is the Ni distribution, which was expected to be homogeneous. Instead, a granular microstructure was revealed. This observed non-uniformity of the Ni layer may be attributed to uneven condensation during thermal evaporation. The high melting point and low vapour pressure of Ni result in a deposition rate and adatom mobility that can be strongly affected by local variations in substrate temperature or vapour flux. Additionally, the annealing process could lead to inhomogeneous Ni recrystallization. This can lead to island-like nucleation and thickness fluctuations across the surface, as previously reported for metal-on-metal thin-film growth [47].

Regarding the Cr distribution, no clear information can be directly extracted from the conventional PIXE maps extracted with OMDAQ 3. However, the new method developed in this work shows (see Figure 3) that it follows the same distribution as gold. Furthermore, in Figure 4, it is possible to observe the inhomogeneous region along the Au fingers, which was previously visible in the 2D gold distribution map shown in Figure 2. However, in this case, the thickness of the gold contacts can also be determined, reaching approximately 300nm, thus eliminating the need for conventional point analysis. It should be clarified that this value does not correspond to the total thickness of the evaporated Au contact (~1 µm) but rather to locally thinner regions associated with the observed inhomogeneities along the Au fingers (see the Au map in Figure 2). This demonstrates the capability of the method to resolve spatial variations in thickness across the sample without requiring localized point measurements.

## 5. Conclusions

In this study, artificial neural networks were successfully applied to analyse simultaneous PIXE and EBS spectra from complex device stacks such as GaSb thermophotovoltaic converters. The method enables non-destructive, depth-resolved 3D elemental mapping, resolving the buried Ni and Cr distributions beneath the Au contacts. It also delivers quantification of local Au thickness and qualitative Ni and Cr distribution with micrometre lateral resolution, directly from the spectral data, without the need for additional point-by-point analysis, thus overcoming the surface bias of conventional 2D IBA mapping.

The approach demonstrated here for one specific case study establishes a path toward automated, standardized workflows for multidimensional material characterization. The methodology is not limited to thermophotovoltaic systems and can be applied to a wide range of multilayer materials, including thin-film coatings, semiconductor heterostructures, and energy devices such as batteries and catalytic systems, where depth-resolved compositional information is critical. By reducing the need for data compression (i.e., 2 × 2 or no compression) and exploiting local spectral correlations through convolutional neural networks, higher effective spatial resolution and richer compositional detail can be achieved from the same experimental datasets.

Future developments will focus on expanding EBS-based sensitivity to light elements such as C, O and N, improving PIXE spectra generation to reduce the dependence on preliminary 2D elemental maps, and integrating complementary signals such as scanning transmission ion microscopy (STIM) to combine density contrast with elemental mapping. Together, these developments will consolidate ion beam analysis as a quantitative, non-destructive tool for 3D elemental characterization applicable to design and optimize the next generation of complex multilayer materials and device interfaces.

## Data Availability

The original contributions presented in this study are included in the article/Supplementary Material. Further inquiries can be directed to the corresponding author.

## Supplementary Materials

The following supporting information can be downloaded at https://www.mdpi.com/article/10.3390/ma19132819/s1: Figure S1: Schematic overview of the ANN-assisted IBA analysis pipeline, from raw listmode data acquisition to 3D elemental reconstruction; Figure S2: (a) EBS spectra: Sum of all EBS spectra over the 130 × 130 μm^2^ analyzed sample represented in a 256 × 256 pixel map; (b) sum of 4 EBS spectra from 4 contiguous pixels; Figure S3: 2D elemental maps with 4 × 4 pixel compression; Figure S4: (a) Position of the five representative PIXE spectra considered to train the network; (b) PIXE spectra for points A, B, C, D and E (shown in the Ni and Au maps). Position of the characteristics x-ray emission are labeled. Raw data were recorded in channels, but the spectra are displayed as a function of energy to facilitate the identification of the X-ray lines; Figure S5: a. Confusion matrix for validation set showing the classification performance of PIXE spectral classes. The strong diagonal pattern indicates high accuracy and minimal misclassification between classes. b. Accuracy as a function of the epochs for training and validation PIXE data; Figure S6: a. Accuracy as a function of the epochs for training and validation of EBS data; b. Confusion matrix for validation set the EBS-class spectral classification model; Table S1: Architecture of the neural network developed for PIXE spectra classification; Table S2: Architecture of the neural network developed for EBS spectra classification; Supplementary Video: 3D-gold.mp4.

## Abbreviations

The following abbreviations are used in this manuscript:

IBA	Ion beam analytical
NM	Nuclear microprobe
PIXE	Particle-induced X-ray emission
EBS	Elastic backscattering spectrometry
XRF	X-ray fluorescence
EDS	Energy-dispersive X-ray spectroscopy
SIMS	Secondary ion mass spectroscopy
TPV	Thermophotovoltaic
ROIs	Regions of interest
STIM	Scanning transmission ion microscopy
