# Supplementary material for: Non-Destructive 3D Elemental Characterization of Multilayer Materials by ANN-Assisted Ion Beam Analysis
Source: Materials (Basel). 2026 Jul 2;19(13):2819. doi: 10.3390/ma19132819 (PMC13363541; doi:10.3390/ma19132819)
Supplement: Supplementary file 1 [file materials-19-02819-s001.zip › materials-4365912-supplementray.pdf]

Article

Non-destructive 3D elemental characterization of multilayer materials by ANN-assisted ion beam analysis

Victoria Corregidor [1,*], Nuno P. Barradas [1,2], Rui C. da Silva [1,3], Teresa Pinheiro [1,4], Carlos Algora [5], Luís C.Alves [1,2]

[1] Departamento de Engenharia e Ciências Nucleares, Instituto Superior Técnico, Universidade de Lisboa, Loures, Portugal;
[2] C2TN, Centro de Ciências e Tecnologias Nucleares, Instituto Superior Técnico, Universidade de Lisboa, Loures, Portugal;
[3] IPFN, Instituto de Plasmas e Fusão Nuclear, Instituto Superior Técnico, Universidade de Lisboa, Portugal;
[4] 4 IBB, Instituto de Bioengenharia e Biociências, Instituto Superior Técnico, Universidade de Lisboa, Portugal;
[5] Instituto de Energía Solar, Universidad Politécnica de Madrid, Avda. Complutense, 30, 28040 Madrid, Spain;
* Correspondence: victoria.corregidor@ctn.tecnico.ulisboa.pt;

Supplementary Material:

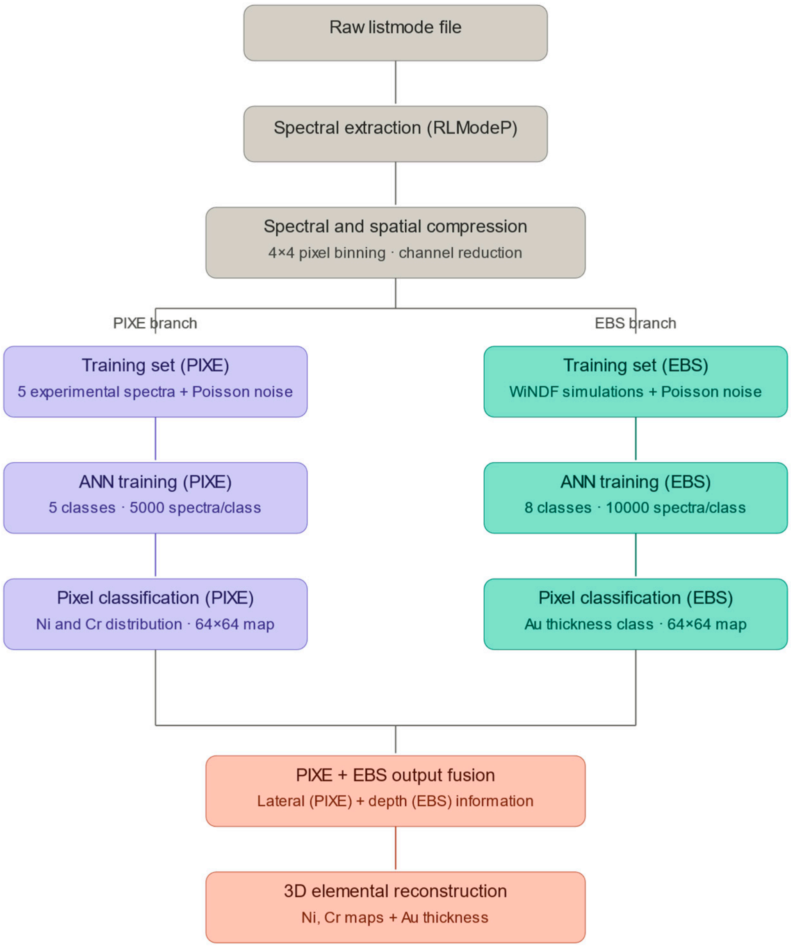

Figure S1. Schematic overview of the ANN-assisted IBA analysis pipeline, from raw listmode data acquisition to 3D elemental reconstruction.

Academic Editor: Firstname Last-name

Received: date
Revised: date
Accepted: date
Published: date

Copyright: © 2026 by the authors. Submitted for possible open access publication under the terms and conditions of the Creative Commons Attribution (CC BY) license.

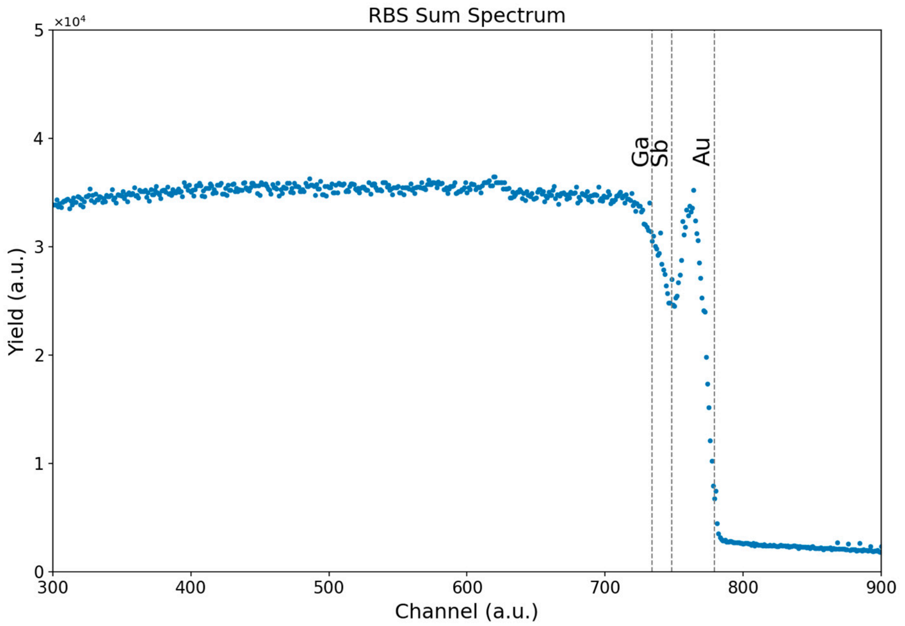

a

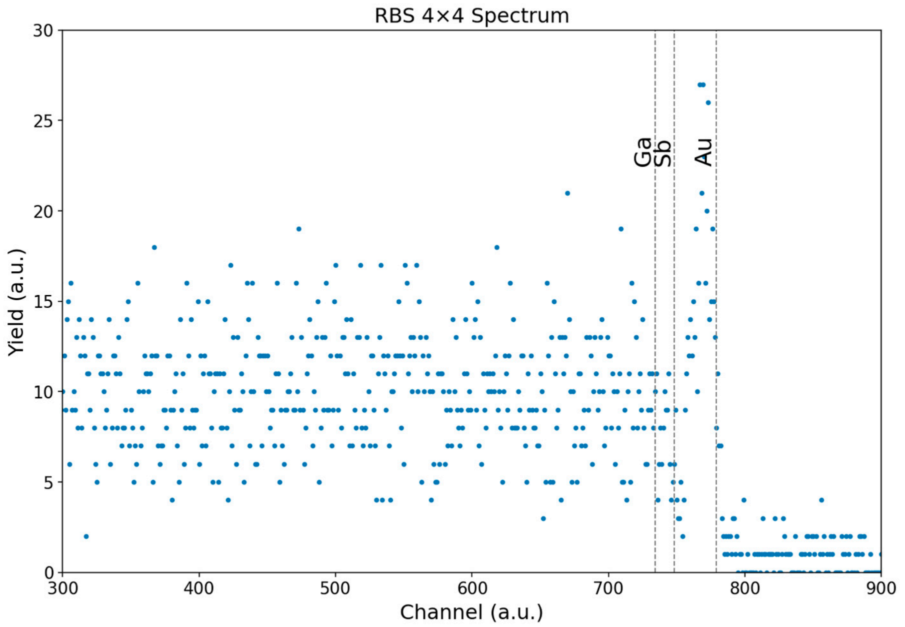

b

Figure S2. a) EBS spectra: Sum of all EBS spectra over the 130x130 μm^2 analyzed sample represented in a 256x256 pixel map; b) sum of 4 EBS spectra from 4 contiguous pixels.

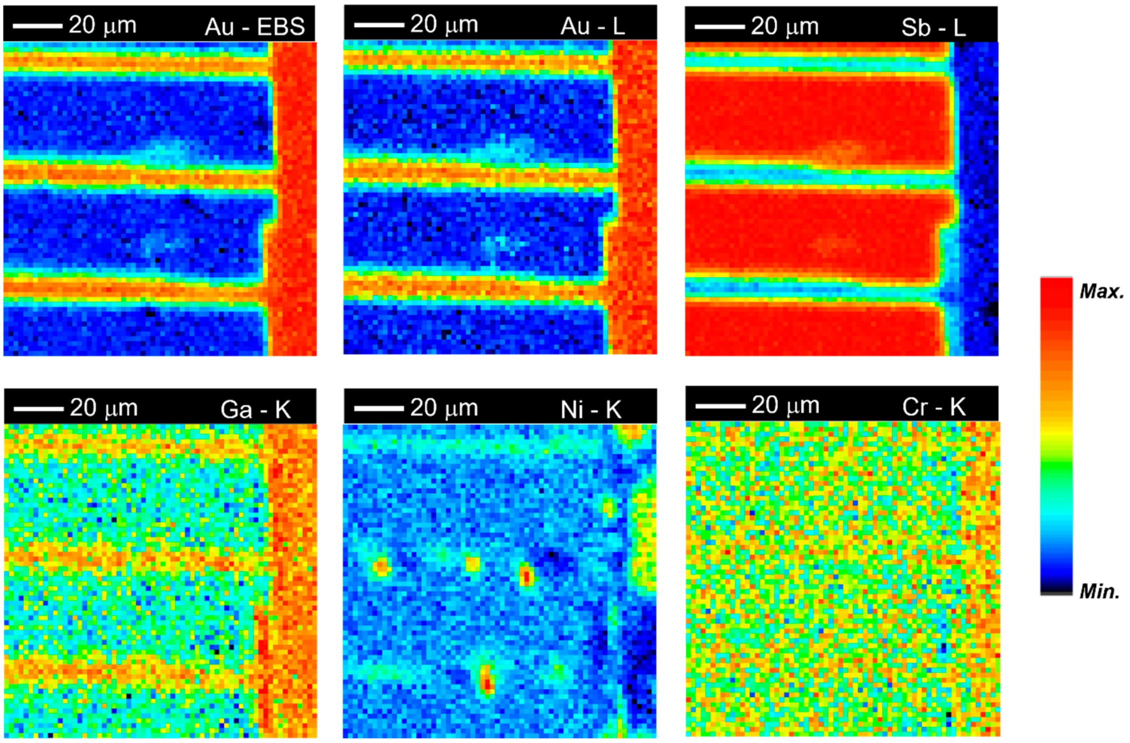

Figure S3. 2D elemental maps with 4 x 4 pixel compression.

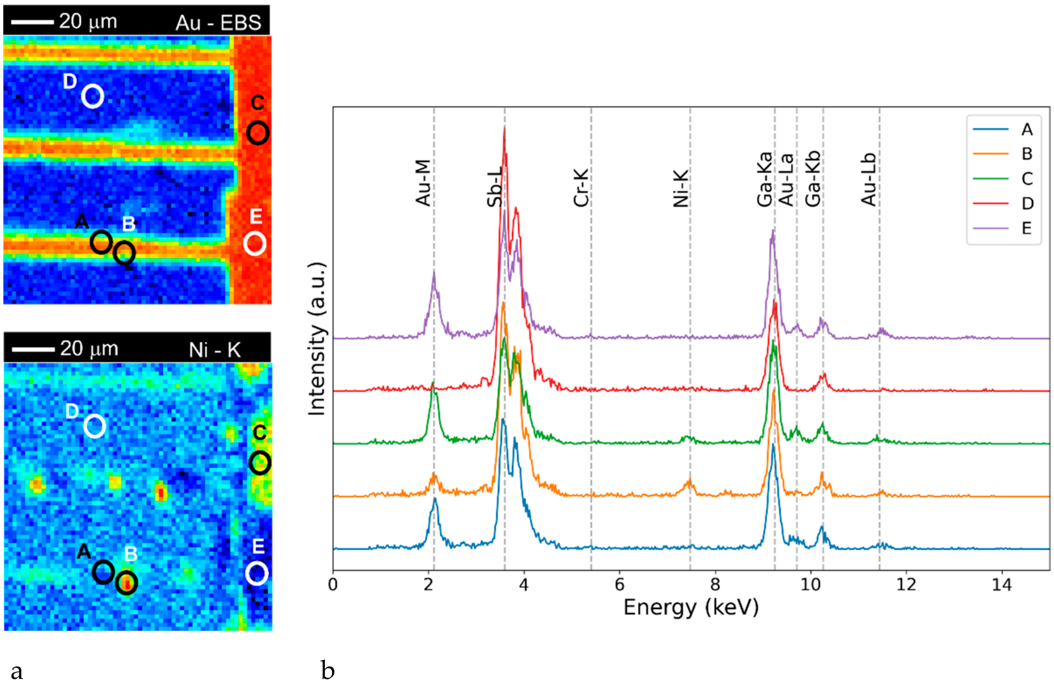

Figure S4. a) Position of the five representative PIXE spectra considered to train the network; b) PIXE spectra for points A, B, C, D and E (shown in the Ni and Au maps). Position of the characteristics x-ray emission are labeled. Raw data were recorded in channels, but the spectra are displayed as a function of energy to facilitate the identification of the X-ray lines.

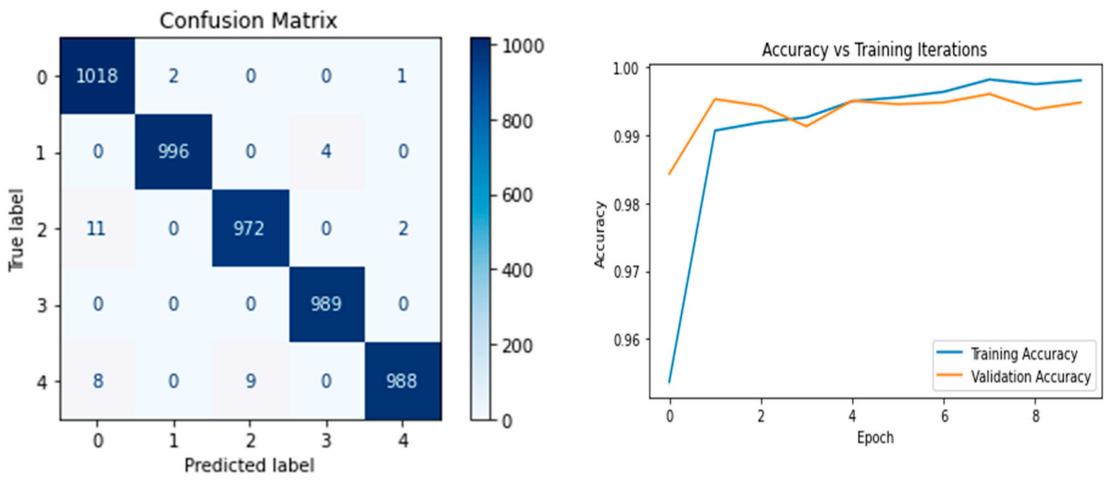

a b

Figure S5. a. Confusion matrix for validation set showing the classification performance of PIXE spectral classes. The strong diagonal pattern indicates high accuracy and minimal misclassification between classes. b. Accuracy as a function of the epochs for training and validation PIXE data.

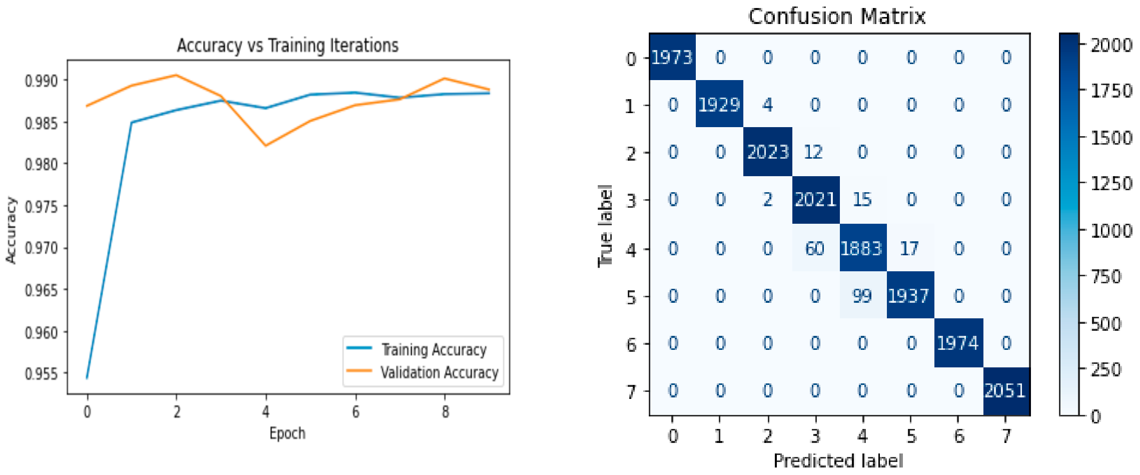

a b

Figure S6. a. Accuracy as a function of the epochs for training and validation EBS data; b. Confusion matrix for validation set the EBS-class spectral classification model.

Table S1. Architecture of the neural network developed for PIXE spectra classification.

model = keras.Sequential()
model.add(layers.Input(shape=(800,)))
model.add(layers.Dense(400, activation='relu'))
model.add(layers.Dense(128, activation='relu'))
model.add(layers.Dense(64, activation='relu'))
model.add(layers.Dense(5, activation='softmax'))
history=model.fit(X_train, y_train, epochs=10, batch_size=32, validation_split=0.2)

Table S2. Architecture of the neural network developed for EBS spectra classification.

model = keras.Sequential()
model.add(layers.Input(shape=(128,)))
model.add(layers.Dense(96, activation='relu'))
model.add(layers.Dense(64, activation='relu'))
model.add(layers.Dense(number_class, activation='softmax'))
model.compile(optimizer='adam', loss='categorical_crossentropy',)
history=model.fit(X_train, y_train, epochs=10, batch_size=32, validation_split=0.2)
